# An Improved UPLC-MS/MS Platform for Quantitative Analysis of Glycerophosphoinositol in Mammalian Cells

**DOI:** 10.1371/journal.pone.0123198

**Published:** 2015-04-10

**Authors:** Laura Grauso, Stefania Mariggiò, Daniela Corda, Angelo Fontana, Adele Cutignano

**Affiliations:** 1 Istituto di Chimica Biomolecolare, Consiglio Nazionale delle Ricerche, via Campi Flegrei 34, 80078 Pozzuoli, Napoli, Italy; 2 Istituto di Biochimica delle Proteine, Consiglio Nazionale delle Ricerche, via Pietro Castellino 111, 80131 Napoli, Italy; Imperial College London, UNITED KINGDOM

## Abstract

The glycerophosphoinositols constitute a class of biologically active lipid-derived mediators whose intracellular levels are modulated during physiological and pathological cell processes. Comprehensive assessment of the role of these compounds expands beyond the cellular biology of lipids and includes rapid and unambiguous measurement in cells and tissues. Here we describe a sensitive and simple liquid chromatography-tandem mass spectrometry (LC-MS/MS) method for quantitative analysis of the most abundant among these phosphoinositide derivatives in mammalian cells, the glycerophosphoinositol (Gro*P*Ins). The method has been developed in mouse Raw 264.7 macrophages with limits of quantitation at 3 ng/ml. Validation on the same cell line showed excellent response in terms of linear dynamic range (from 3 to 3,000 ng/ml), intra-day and inter-day precision (coefficient of variation ≤ 7.10%) and accuracy (between 98.1 and 109.0%) in the range 10-320 ng/ml. As proof of concept, a simplified analytical platform based on this method and external calibration was also tested on four stimulated and unstimulated cell lines, including Raw 264.7 macrophages, Jurkat T-cells, A375MM melanoma cells and rat basophilic leukemia RBL-2H3 cells. The results indicate a wide variation in Gro*P*Ins levels among different cell lines and stimulation conditions, although the measurements were always in line with the literature. No significant matrix effects were observed thus indicating that the here proposed method can be of general use for similar determinations in cells of different origin.

## Introduction

The phosphoinositides are structural and functional components of cell membranes and precursors of signalling mediators [1]. Among their water-soluble derivatives, the glycerophosphoinositols, the products of phospholipase A_2_ IVα activity, play key roles in several physiological processes including inflammation and remodelling of the actin cytoskeleton [2–4]. The most abundant and ubiquitous member of this family is the glycerophosphoinositol (Gro*P*Ins), whose intracellular levels are modulated during cell proliferation, differentiation, or oncogenic transformation [2, 5–9]. The role of Gro*P*Ins in orchestration of signalling cascades in both physiological and pathological settings is strictly dependent on its cellular concentration, thus full understanding of the biological function of this chemical mediator mandatorly requires its accurate quantitation. Gro*P*Ins is a charged metabolite that tends to undergo ionic changes that challenge the use of chromatographic methods for the analysis of this phosphoinositide-derived metabolite in biological samples. In particular, quantitative analysis by reversed-phase chromatography typically suffers from poor retention and peak shape of a charged analyte, whereas anion-exchange chromatography is limited by low selectivity [10, 11]. Few liquid chromatography-mass spectrometry (LC-MS) methods have been proposed to overcome these technical drawbacks without fully complying with the demand of simplicity and accuracy for a general use of the analytical procedures [12–14]. Only recently, Patton-Vogt and coworkers have introduced the use of hydrophilic interaction liquid chromatography (HILIC) that seems to provide a valid approach to analyze this charged molecule and related compounds in biological samples [15].

Here we report a significant improvement of the hydrophilic interaction-based separation by using Ultra Performance Liquid Chromatography (UPLC) that increases speed, resolution and sensitivity compared to standard HPLC methods. Specificity of Gro*P*Ins detection is achieved by multiple reaction monitoring (MRM) on a triple quadrupole mass spectrometer (MS) with electrospray ionization (ESI) in negative polarity. The UPLC-MS method has been tested on mouse Raw 264.7 macrophages as matrix, a cell line already investigated for quantitive measurements of Gro*P*Ins [3], and successfully applied to evaluate the content of Gro*P*Ins in four different mammalian cells, including the above mentioned mouse Raw 264.7 cell line, rat basophilic leukemia RBL-2H3 cells, human metastasizing melanoma A375MM cells and human Jurkart T-cells, both at the basal level and after different treatments.

## Experimental

### Materials

Gro*P*Ins was kindly provided by Euticals S.p.a. (Lodi, Italy). Inositol-*d*_*6*_, ammonium hydroxide solution (≥ 25% in water, eluent additive for LC-MS), fatty-acid-free bovine serum albumin (faf BSA), and lipopolysaccharides (LPS) from *E*. *coli* 055:B5 were obtained from Sigma-Aldrich (St. Louis, MO, USA). HPLC grade water was obtained from VWR (Leuven, Belgium). HPLC grade acetonitrile and methanol were purchased from Merck (Darmstadt, Germany). Super purity acetic acid was purchased from Romil (Cambridge, United Kingdom). For cell-growth-media composition: MEM, DMEM, DMEM-F12, RPMI Medium 1640, foetal bovine serum were all from Gibco by Life Technologies (Life Technologies Italia, MB, Italy); penicillin, streptomycin and L-glutamine were from Sigma-Aldrich (St. Louis, MO, USA). All other cell culture reagents were of the highest purity and obtained from Gibco BRL (Grand Island, NY, USA).

### Standard solutions

Gro*P*Ins was dissolved in water and aliquoted at a final concentration of 2 mg/ml. Working standard solutions of Gro*P*Ins (3–3,000 ng/ml) were obtained by dilution of the stock solution in water and stored at -20°C until use. Inositol-*d*_*6*_ (internal standard, IS) was dissolved in water to a final concentration of 2 mg/ml; a working standard solution of 200 μg/ml was prepared by dilution of stock solution with water and stored at -20°C until use.

### Cell Culture and Sample Preparation

Mouse Raw 264.7 macrophages were bought by the Corda's laboratory in 2003, from the American Type Culture Collection (ATCC catalogue number: TIB-71). The cells were maintained in DMEM supplemented with 10% heat-inactivated (30 min at 55°C) foetal bovine serum, 100 U/ml penicillin, 0.1 mg/ml streptomycin, 2 mM L-glutamine [16]. Raw 264.7 cell extracts were obtained under normal growth condition or after LPS-stimulation (20 μg/ml, in growth medium, 30 min at 37°C).

Human lymphoma Jurkat T-cells [17] were maintained in RPMI Medium 1640 supplemented with 10% heat-inactivated foetal bovine serum, 100 U/ml penicillin, 0.1 mg/ml streptomycin, 2 mM L-glutamine [18]. Jurkat T-cell extracts were obtained under basal conditions or after ionomycin-treatment (10 μM, in plain RPMI Medium 1640 plus 1% faf BSA, 15 min at 37°C).

Rat basophilic leukemia (RBL-2H3) cells were bought by the Corda's laboratory in 2003, from the ATCC (ATCC catalogue number: CRL-2256). The cells were maintained in MEM supplemented with 15% heat-inactivated foetal bovine serum, 100 U/ml penicillin, 0.1 mg/ml streptomycin, 2 mM L-glutamine [3]. RBL-2H3 cell extracts were obtained under basal conditions and after ionomycin-treatment (10 nM, in plain MEM, 15 min at 37°C).

Human metastasizing melanoma A375MM cells, obtained from the Institute of Oncological Research (IRO) in Barcelona through the Egea laboratory at the Barcelona University [19], were maintained in DMEM/F12 (1:1) supplemented with 10% foetal bovine serum, 100 U/ml penicillin, 0.1 mg/ml streptomycin, 2 mM L-glutamine [18]. A375MM cell extracts were obtained under basal conditions and after ionomycin-treatment (10 nM, in plain DMEM-F12, 15 min at 37°C).

Gro*P*Ins extraction was performed as previously reported [2, 20] with minor modifications. Briefly, all the incubations were terminated by aspiration of the medium, washing twice with 0.9% NaCl (4° C), and adding 6 ml of -20°C methanol/ 1 M HCl (1:1) with a two-phase extraction carried out by addition of a half volume of chloroform. After rapid vigorous mixing, the extractions were allowed to settle under gravity, and then the aqueous (upper) and organic (lower) extraction phases were separated. The upper (aqueous) phase was aliquoted (1.5 ml) for lyophilising and resuspension for mass analysis. Each cell line was assessed by replicate analysis from at least two independent petri dishes of each cell type. In parallel, an additional petri dish, equivalent to that undergoing mass analysis, was used for blind-cell counting in a Neubauer chamber, after exaustive cell detachment. For cell volume measurements, cells were grown to near confluence in their respective medium and then detached (when necessary), washed, and resuspended in 0.9% NaCl solution. Samples were used to measure relative cell volumes in comparison with Raw 264.7 cells by flow cytometry. The Raw 264.7 cell volume was taken as 0.77 ±0.03 pl [3].

### LC-MS conditions

Chromatographic separations were achieved by a Waters (Milford, MA, USA) ACQUITY UPLC BEH Amide column (100 x 2.1 mm, 1.7 μm) at 30°C on a Waters ACQUITY UPLC System. Eluent A: acetonitrile and eluent B: ammonium hydroxide (0.01%, adjusted to pH 9.0 using acetic acid)-acetonitrile (95:5, v/v). The elution program consisted of an isocratic elution at 7.5% eluent B for 1 min, followed by a gradient from 7.5 to 52.5% B in 2.3 min for a total elution time of 3.3 min. The column was equilibrated for 3.2 min at 7.5% B prior to the next analysis. The injection volume was 2 μl. Gro*P*Ins and internal standard were eluted at a peak retention time of 2.62 min and 3.15 min respectively, with a flow rate of 0.6 ml/min.

Mass spectrometry (MS) analysis was carried out on API 3200 Triple Quadrupole (ABSciex, Foster City, CA, USA) equipped with a Turbo V Ion Spray Source used in negative-ion mode. Quantitation was achieved monitoring the [M-H]^-^ ion at *m/z* 332.9 in the first quadrupole and product ions at *m/z* 152.9 and *m/z* 241.0 in the third quadrupole. For IS, transition at *m/z* 185.1→167.0 was selected.

The following source parameters were used: curtain gas (N_2_) at 25 psi, ion source gas (GS1) at 55 psi, turbogas (GS2) at 55 psi, desolvation temperature at 550°C, collision activated dissociation gas (CAD) at 5 a.u. and ion-spray voltage at -4500 V. Gro*P*Ins standard (20 ng/ml) optimized mass spectrometry parameters: declustering potential (DP) at -50 V, entrance potential (EP) at -11 V, collision energy (CE) at -23 eV (for *m/z* 332.9→241.0) and at -31 eV (for *m/z* 332.9→152.9), cell exit potential (CXP) at -3 V. Inositol-*d*_*6*_ (50 ng/ml) optimized mass spectrometry parameters: DP = -35 V, EP = -11 V, CE = -21 eV, CXP = -2.5 V. The dwell time was set to achieve a total scan time of 0.33 s. The autosampler cooler was maintained at 10°C. Analyst software (version 1.5.2; ABSciex) was used for data recording and Multiquant software (version 2.0.2; ABSciex) for quantitative analyses.

### Method Validation

The LC-MS/MS method was validated through evaluation of specificity, linearity, intra- and inter-day precision and accuracy in accordance with the currently-approved FDA guidelines for the validation of bioanalytical methods [21]. Each analytical run consisted of blank samples (water without analyte and without IS), a zero sample (matrix with IS), calibration standards at different concentration levels (in water and in matrix, with IS), QC samples (samples different from calibration standards with known concentration of analyte and IS in water).

#### Specificity and selectivity

Specificity was evaluated on the basis of the ion transition at *m/z* 332.9→152.9 in spiked samples, cell extract matrix and standard samples in water. Results were compared to those obtained with the second mass transition at *m/z* 332.9→241.0 to assess the selectivity of detection. Chromatographic and spectrometric parameters were optimized to minimize the interference on the analyte from endogenous substances and to obtain the highest signal to noise ratio.

#### Linearity

Seven concentration levels of Gro*P*Ins standard dissolved in water (3, 10, 30, 100, 300, 1,000 and 3,000 ng/ml) were analyzed in triplicate. The average of five measurements was used for building up the calibration curve. A known amount of IS (1 μg/ml) was spiked at all calibration points. Peak area ratios of Gro*P*Ins/ IS were used to express the Gro*P*Ins response. Limit of quantitation (LOQ) and limit of detection (LOD) were both established in water. The linearity of the calibration curve was also verified in the cell extract matrix (n = 5), by spiking known amount of Gro*P*Ins (10, 20, 40, 80, 160, 320 ng/ml) and a constant amount of IS (1 μg/ml) and compared with the same concentration range of Gro*P*Ins in water.

#### Intra-day and inter-day assay for precision and accuracy

Intra- and inter-day precision and accuracy were determined through the analysis of five replicates at five different concentrations of spiked Gro*P*Ins within the same day and for three different days in the cell extract matrix as above indicated. The samples were prepared according to the procedure mentioned before. The precision was expressed as coefficient of variation (CV, [standard deviation/ measured mean concentration] x 100), while the accuracy was expressed as [measured mean concentration/ nominal concentration] x 100.

#### Matrix effect

Cell samples were extracted according to the procedure reported above. All but one samples were spiked with Gro*P*Ins at five different concentrations. Extracts were analyzed by LC-MS/MS under the same experimental conditions and at the same concentration levels as those used for Gro*P*Ins spiked in water. A constant amount of IS (1 μg/ml) was added to all samples. The matrix effect was evaluated comparing the responses of Gro*P*Ins in matrix and in water. Matrix effects (%) were calculated as [(peak area from the spiked sample-peak area from the non-spiked sample)/ peak area of standard sample] x 100. Results are expressed as the mean of five measurements at five calibration points.

## Results and Discussion

**Fig 1:**
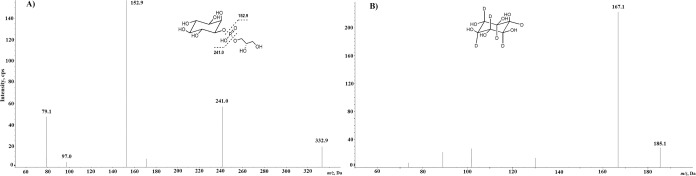
MS/MS spectra. (A) Gro*P*Ins. (B) inositol-*d6* (IS).

In order to optimize the MS conditions, a full-scan ESI mass spectrum in negative-ion mode was recorded by direct infusion of a water solution of Gro*P*Ins standard. The MS/MS spectrum (Fig 1A) showed a main peak [M-H]^-^ at *m/z* 332.9 for the molecular species and four characteristic fragments at *m/z* 241.0 [M-glycerol]^-^, *m/z* 152.9 [M-inositol]^-^, *m/z* 97.0 [H_2_PO_4_^-^] and *m/z* 79.1 [HPO_3_^-^]. The first two fragmentations were selected for the MRM analysis. In particular, the transition due to the loss of inositol (332.9→152.9) was used for quantification of Gro*P*Ins by LC-MS on an ACQUITY BEH-amide column, whereas the peak area ratio between the first (332.9→152.9) and the second transition (332.9→241.0) was monitored for the assessment of structural specificity. Deuterated analogues of Gro*P*Ins are not commercially available, thus we selected inositol-*d*_*6*_ as internal standard (IS) and a single mass transition (185.1→167.1) was used (Fig 1B). It is to note that the absence of isotopically labelled analogs of Gro*P*Ins is one of the major technical hurdles in the analysis of this class of lipid mediators. In other analytical approaches [15], compounds with chemical property and behaviour similar to Gro*P*Ins have been already used as Internal Standard.

To optimize the chromatographic performance and to avoid the occurrence of tailing peaks we tested several combinations of methanol and acetonitrile with aqueous buffers at different pH and temperatures. The best result was obtained with a gradient of increasing amount of ammonium hydroxide (0.01%, adjusted to pH 9 using acetic acid) in acetonitrile at 30°C (Experimental section). In the experimental conditions Gro*P*Ins and IS eluted with a slightly different retention time. However, although the ionization and chromatographic responses were not identical to those of the target analyte, inositol-*d*_*6*_ gave satisfactory reliability in the present study. In fact, the instrumental response measured as Gro*P*Ins/ IS peak area ratio fitted a linear regression over a range of Gro*P*Ins concentrations between 3 ng/ml and 3 μg/ml, with a correlation coefficient (r^2^) always higher than 0.9991.

**Fig 2:**
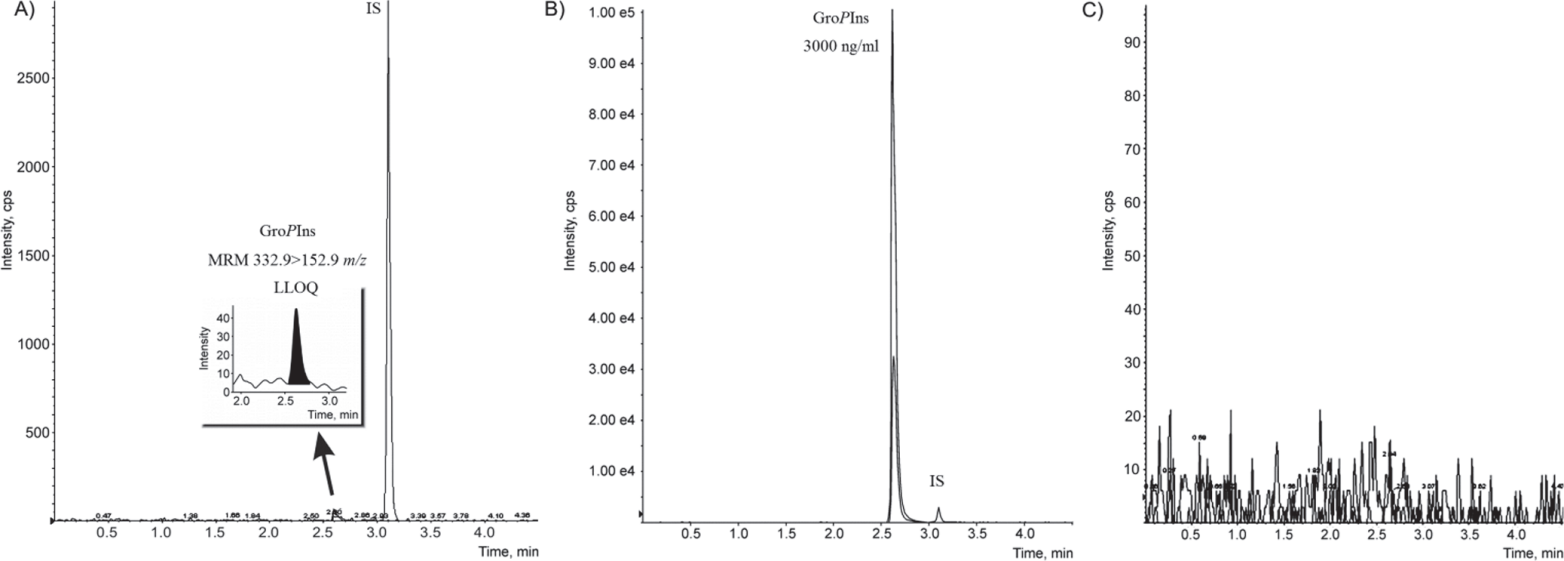
Representative LC-MRM chromatograms. (A) LOQ (3 ng/ml) of Gro*P*Ins in water. (B) Gro*P*Ins at the highest standard concentration (3,000 ng/ml) in water. (C) Blank sample after injection of the highest concentration of Gro*P*Ins (3,000 ng/ml).

The new protocol allowed detection of this lipid derivative at micromolar concentration (LOD was at 1 pg) with a LOQ of 3 ng/ml (S/N ≥10) (Fig 2A). More interestingly, no carry-over effect was detected during analysis of a blank sample even after injection of a standard sample at the highest calibration point (3 μg/ml) (Fig 2B and 2C).

**Fig 3:**
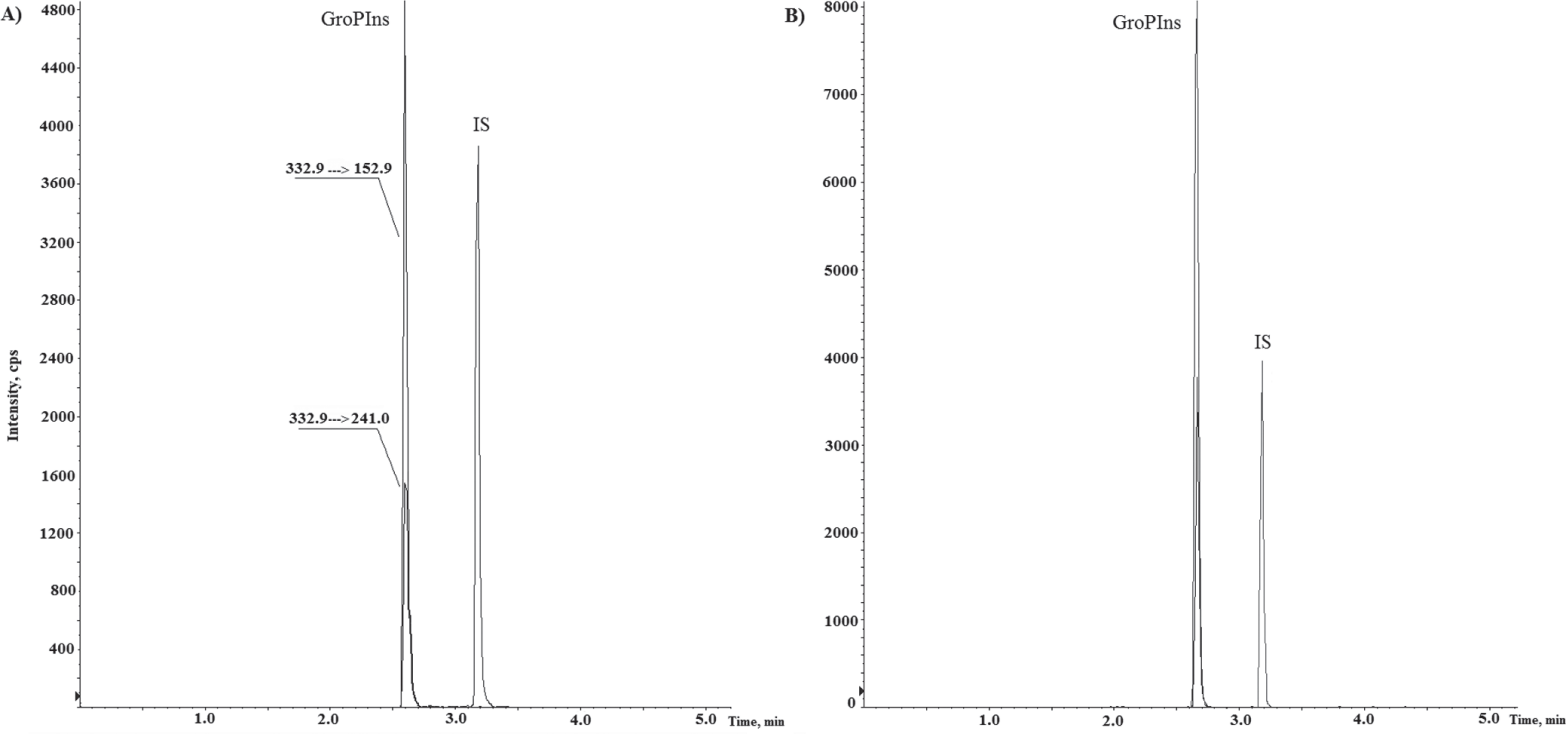
Representative LC-MRM chromatogram of Gro*P*Ins and of the IS. (A) Cell extract of Raw264.7. (B) Cell extract of Raw 264.7 spiked with standard Gro*P*Ins.

A crucial point in mass analyses of biological samples is the potential interference of matrix components on detection of the analyte of interest, with signal suppression/ enhancement due to co-eluting compounds. One of the most effective way to eliminate matrix effects in quantitative analysis is represented by the matrix-matched calibration method, which consists in spiking the blank matrix with increasing amount of standard at the desired calibration points. Since Gro*P*Ins has been detected in all eukaryotic cells at basal level and in absence of an analyte-free matrix, all but one samples had to be spiked with the standard at different concentrations and the analyte concentration is obtained by back-calculation, extrapolating the x-intercept of the linear curve, according to a procedure known as standard addition calibration method. In this way, after addition of inositol-*d*_*6*_ as internal standard, concentration of Gro*P*Ins was measured in human Raw 264.7 macrophages that have been subject of previous studies for the presence of this water-soluble metabolite [2, 3]. Replicate analyses of these extracts showed mean levels of this lipid derivative of 122.34 ±3.85 μM in unstimulated cells, which was consistent with the literature [3]. A typical chromatogram representing a real sample of Raw 264.7 cell extract with basal level of Gro*P*Ins (88.4 ng/ml) and the cell extract spiked with 80ng/ml of Gro*P*Ins standard is shown in Fig 3.

**Fig 4:**
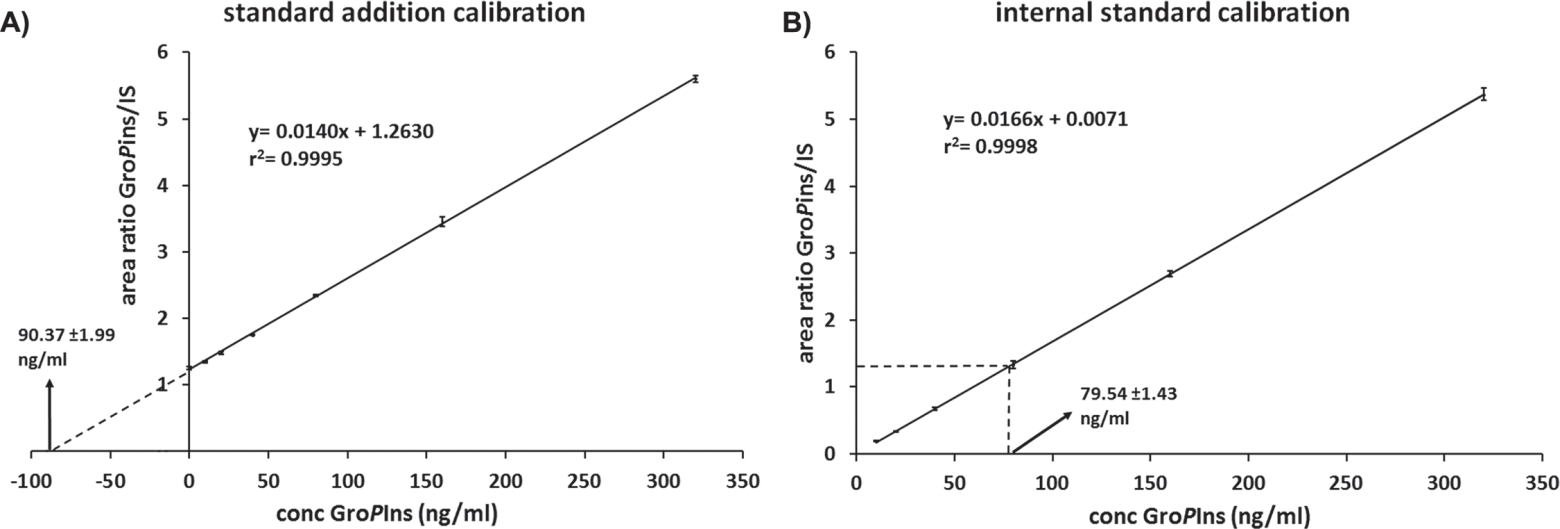
Linearity of Gro*P*Ins and analyte concentration in Raw 264.7 cell extract. (A) Standard addition calibration in matrix. (B) Internal standard calibration in water. Error bars are expressed as SD.

The analyses were repeated three times (n = 5) and the curves exhibited excellent linearity (Fig 4), without any significant matrix effect (<15%, Table 1). Furthermore, the area ratio of the peaks corresponding to the two MRM transitions at *m/z* 332.9→152.9 and *m/z* 332.9→241.0 remained constant with an average value of 2.55 throughout all analyses, including biological extracts, matrix-matched samples and pure Gro*P*Ins standard in water, thus corroborating the absence of interference from endogenous substances.

**Table 1:** Matrix effect in Raw 264.7, RBL-2H3 and Jurkat T-cell extracts.

		Matrix effect (% ± SD)^a^	
Gro*P*Ins spiked conc. in matrix (ng/ml)	Raw 264.7	RBL-2H3	Jurkat
**10**	86.94 ±0.62	104.31 ±2.90	102.32 ±6.02
**20**	85.59 ±0.05	112.44 ±2.19	105.34 ±1.84
**40**	87.55 ±2.01	113.70 ±1.09	107.93 ±2.54
**80**	86.94 ±1.59	113.47 ±0.94	111.03 ±1.33
**160**	86.59 ±1.05	109.10 ±3.13	114.87 ±0.11

^a^Each point is based on five replicates.

Finally, the reliability of this UPLC-MS method was tested by measuring intra-day and inter-day accuracy and precision (n = 5) of Gro*P*Ins spiked to Raw 264.7 macrophages at concentration ranging from 10 to 320 ng/ml. As reported in Table 2, the analysis proved the accuracy and precision of the analytical platform that showed deviation from the true value within 10%, and a coefficient of variation between 1.96 and 7.10%.

**Table 2:** Intra-day and inter-day precision and accuracy in Raw 264.7 cell extract.

Gro*P*Ins	Intra-day^a^						Inter-day^b^					
**Nominal concentration (ng/ml)**	10	20	40	80	160	320	10	20	40	80	160	320
**Mean**	10.90	20.44	40.26	80.14	161.74	313.96	10.48	20.64	40.30	81.16	160.14	315.12
**Precision (CV %)** ^**c**^	3.58	3.16	2.71	4.69	2.11	1.96	7.10	4.81	3.49	3.44	2.43	2.88
**Accuracy (%)** ^**d**^	109.0	102.2	100.6	100.2	101.1	98.1	104.8	103.2	100.8	101.4	100.1	98.5

^a^n = 5.

^b^n = 3 days with five replicates per day.

^c^[Standard deviation/ measured mean concentration] x 100.

^d^[Measured mean concentration/ nominal concentration] x 100.

Because the data on Raw 264.7 macrophages showed a good linearity and absence of matrix effect we attempted to further improve the methodology by using an external calibration curve in water. In fact, although the standard addition method allows to overcome interferences due to matrix components, it is time consuming and requires a large number of measurements per sample. When significant matrix effect can be excluded, it is much easier to achieve quantitation against a calibration curve prepared in water. Therefore we compared the Gro*P*Ins response of the same cell extract of Raw 264.7 macrophages obtained by both standard addition calibration and internal standard calibration in water.

As showed in Fig 4, the two methods gave comparable results (difference below 15%) suggesting that the use of a calibration method in water is a suitable approach for measuring this chemical mediator.

It is worth noting that when extended to different biological matrices the analytical method still showed reliable parameters in terms of linearity (Table 3), matrix effect (Table 1) and intra-day and inter-day precision and accuracy (Tables 4 and 5). With these results in our hands we measured, by using an external calibration in water, the cellular concentration of Gro*P*Ins in four different cell lines, namely Raw 264.7 macrophages, rat basophilic leukemia RBL-2H3 cells, human metastasizing A375MM melanoma cells and Jurkat T-cells, all relevant in studies on Gro*P*Ins function [2, 3].

**Table 3:** Linearity and Gro*P*Ins concentration in RBL-2H3 and Jurkat T-cell extracts by internal standard calibration and standard addition method.

	RBL-2H3 cells^a^		Jurkat T-cells^a^	
	Internal Standard calibration	Standard Addition calibration	Internal Standard calibration	Standard Addition calibration
**Gro*P*Ins conc. range (ng/ml)**	10–160	10–160	10–160	10–160
**Slope**	0.0244 ±0.0021	0.0359 ±0.0023	0.0244 ±0.0021	0.0237 ±0.0068
**Intercept**	0.0718 ±0.0165	0.7745 ±0.0450	0.0718 ±0.0165	0.5145 ±0.0356
**Coefficient of correlation (r** ^**2**^ **)**	0.9994 ±0.0003	0.9993 ±0.0001	0.9994 ±0.0003	0.9992 ±0.0007
**Gro*P*Ins conc. (ng/ml)**	22.04 ±2.44	21.56 ±1.25	19.99 ±2.72	21.67 ±1.77

^a^Analyses were performed in triplicate (n = 5). Values are expressed as mean ± SD.

**Table 4:** Intra-day precision and accuracy in RBL-2H3 and Jurkat T-cell extracts.

Gro*P*Ins	RBL-2H3 cells					Jurkat T-cells				
**Nominal concentration (ng/ml)**	10	20	40	80	160	10	20	40	80	160
**Mean** ^**a**^	10.21	20.57	41.42	78.49	158.73	9.76	18.32	38.76	81.61	157.07
**Precision (CV%)** ^**b**^	7.45	7.60	3.91	2.90	1.93	7.39	5.87	7.37	1.73	0.46
**Accuracy (%)** ^**c**^	102.1	102.9	103.6	98.1	99.2	97.6	91.6	96.9	102.0	98.1

^a^n = 5.

^b^[Standard deviation/ measured mean concentration] x 100.

^c^[Measured mean concentration/ nominal concentration] x 100.

**Table 5:** Inter-day precision and accuracy in RBL-2H3 and Jurkat T-cell extracts.

Gro*P*Ins	RBL-2H3 cells					Jurkat T-cells				
**Nominal concentration (ng/ml)**	10	20	40	80	160	10	20	40	80	160
**Mean** ^**a**^	9.30	21.74	44.75	78.35	158.83	9.75	19.08	40.37	82.73	158.34
**Precision (CV %)** ^**b**^	14.79	12.12	11.80	4.61	2.24	8.53	10.71	8.43	8.83	3.57
**Accuracy (%)** ^**c**^	93.0	108.7	111.9	97.9	99.3	97.5	95.4	100.9	103.4	99.0

^a^n = 3 days with five replicates per day.

^b^[Standard deviation/ measured mean concentration] x 100.

^c^[Measured mean concentration/ nominal concentration] x 100.

In agreement with the experimental design reported above, Gro*P*Ins was assessed in the above mammalian cell lines at basal level and after treatment with stimuli, such as the proinflammatory lipopolysaccharide (LPS) or the calcium ionophore ionomycin, which are both known to trigger the synthesis of this chemical mediator through the activation of phospholipase A_2_Ivα [2,3]. Intracellular levels of Gro*P*Ins in unstimulated Raw 264.7 macrophages and Jurkat T-cells were reported to be 115 ±9 μM and 45 ±1 μM, respectively [3]. These values were fully consistent with those assessed by application of this new UPLC-MS method (Table 6). In addition, we also recorded an increase of about 1.6 fold in Gro*P*Ins levels in Raw 264.7 macrophages after stimulation with 20 μg/ml LPS (Table 3), which was also in agreement with previously reported data [16]. Human metastasizing A375MM melanoma cells show relatively high concentrations of Gro*P*Ins, in line with our reported data on several transformed cell lines [2, 9]. In these systems, ionomycin was able to slightly increase the Gro*P*Ins levels whereas this ionophore did not affect the Gro*P*Ins levels in Jurkat T-cells and RBL-2H3 cells. These results are in agreement with the literature about Jurkat T-cells [4], confirming that the activation of PLA_2_IVα in this system is generally not evident, possibly due to basal low levels of arachidonoyl-phosphoinositides, the Gro*P*Ins precursors, or to the involvement of a Ca^2+^-independent enzyme in Gro*P*Ins production. In RBL-2H3 cells, differently from what previously reported in these cells stimulated by the Ca^2+^ ionophore A23187 [3], we could not measure any increase in the Gro*P*Ins level. It should be noticed however that the ionomycin nanomolar concentrations used in this cell system was determined by a cell-specific toxicity of micromolar range of this ionophore. This suggests that in these cells the calcium-concentration increase was not high enough to elicit the activation of PLA_2_IVα. These latter results require further investigation, that however is beyond the aim of the present study.

**Table 6:** Gro*P*Ins levels in untreated and stimulated cells measured by external calibration method.

		Raw 264.7		Jurkat T-cell		RBL-2H3		A375MM	
**Cell vol. (pl)**		0.77 ±0.12		0.77 ±0.09		0.79 ±0.08		1.24 ±0.16	
**(mean ±SD)**							
**Treatment**		unstim.	LPS	unstim.	ionomycin	unstim.	ionomycin	unstim.	ionomycin
**(15 min, 37°C)**			(20 μM)		(10 μM)		(10 nM)		(10 nM)
**Gro*P*Ins**	**#1**	110.65 ±4.32	186.02 ±3.40	32.53 ±1.10	31.31 ±1.59	44.46 ±6.64	45.39 ±1.74	662.08 ±44.22	708.10 ±9.67
**(μM ±SD)** ^**a**^	**#2**	127.41 ±6.11	204.90 ±1.34	34.66 ±1.08	37.93 ±2.09	55.60 ±6.01	47.71 ±2.38	673.95 ±28.27	754.47 ±21.17
**Recovery (%)**		86.0 ±4.9		76.3 ±6.1		81.1 ±3.8		85.2 ±6.6	

^a^Gro*P*Ins mean concentration (n = 6) from biological experiments in duplicate (#1 and #2). *unstim*., unstimulated; *vol*., volume.

The overall extraction recovery was very high with all the cell lines (Table 6), thus proving that the whole protocol is also suitable for analysis of this metabolite when there is little availability of biological sample.

## Conclusions

Assessment of Gro*P*Ins in different cell lines was successfully achieved by UPLC-triple quadrupole mass spectrometry method with a BEH Amide column for chromatografic elution. Identification and quantitation of the analyte is based on MRM specific transition at *m/z* 332.9→152.9. The robustness of the method was proven by the results obtained in tests of linearity range, matrix effect and intra-day/ inter-day reproducibility. The short time (3.3 min) of the UPLC chromatographic run significantly reduces the time of analysis, making the whole process faster and allowing the evaluation of larger numbers of samples. This is a major advantage when comparing this protocol to the previously reported HPLC-based methods. Measurements by external calibration also allowed evaluation of minimum differences in Gro*P*Ins levels in mammalian cells within a wide rage of concentrations and under different physiological conditions. The success of this approach is of general interest and suggests that the analytical platform based on UPLC-BEH Amide column could be extended to both molecules chemically related to Gro*P*Ins and different biological targets, such as other cells of the immune systems that represent attractive candidates to study the physiological role of Gro*P*Ins.
